# The relationship between work-related outcomes and symptoms in early breast cancer survivors receiving adjuvant endocrine therapy

**DOI:** 10.1016/j.apjon.2022.01.003

**Published:** 2022-01-21

**Authors:** Mayumi Nakao, Hiroko Komatsu, Tetsu Hayashida, Maiko Takahashi, Tomoko Seki, Kaori Yagasaki

**Affiliations:** a Keio University Graduate School of Health Management Course for Nursing, Kanagawa, Japan; b Japanese Red Cross Kyushu International College of Nursing, Munakata, Japan; c Department of Surgery, Keio University School of Medicine, Tokyo, Japan; d Keio Cancer Center, Keio University School of Medicine, Tokyo, Japan; e Faculty of Nursing and Medical Care, Keio University, Kanagawa, Japan

**Keywords:** Breast cancer survivors, Endocrine therapy, Symptoms, Work

## Abstract

**Objective:**

This study examined the relationship between symptom burdens and work-related outcomes, including work participation and overall work impairment (OWI) among breast cancer survivors (BCS) receiving adjuvant endocrine therapy (AET).

**Methods:**

This was a cross-sectional study with 140 BCS of working age receiving AET. Data were collected using self-report questionnaires that included an assessment of symptoms and their employment status, and OWI. Data were analyzed using descriptive statistics and multiple logistic regression analysis.

**Results:**

A total of 111 (79%) survivors reported being employed at the time of the survey. Symptom burdens were not associated with unemployment. Of the 110 working BCS receiving AET, symptom burdens were significantly related to a higher degree of OWI (OR ​= ​2.14, 95% CI, 1.58–2.89, *P* ​≤ ​0.001).

**Conclusions:**

Participating BCS receiving AET continued to work while experiencing symptoms, with survivors who experienced high symptom burdens being negatively affected in their work life. Healthcare providers need to assess and manage symptoms and their impact on work, with the help of employers, to improve the quality of work life of BCS receiving AET.

## Introduction

In 2020, the estimated number of new breast cancer cases was greater than 2.2 million worldwide, with about 67% belonging to a working age.^1^ A five-year prevalence of breast cancer is highly regular and is estimated at 7.79 million cases.^1^ A 5–10 year period of adjuvant endocrine therapy (AET) is recommended among women diagnosed with hormone receptor-positive breast cancer.^2^ The number of survivors, who work with unique support needs, associated with receiving AET is probable to increase.

Commonly reported symptoms include vasomotor symptoms (including hot flashes and night sweats), musculoskeletal symptoms (including joint pain), cognitive symptoms (including concentration and memory problems), sleep disturbances, gynecological symptoms, fatigue, and distress (including depression and anxiety).^3^, ^4^, ^5^ In general, endocrine therapy is more tolerable than chemotherapy and radiotherapy. However, these symptoms can negatively affect the quality of life (QOL) of a number of survivors because of the long-term treatment. A recent study identified that endocrine therapy has a persistently negative and clinically significant impact on patients’ QOL, multiple domains of functioning, and their symptoms, while the effects of chemotherapy are highly transient and restricted, with no impact on patients’ QOL at two years postdiagnosis.^6^ Additionally, qualitative studies have concluded that the associated symptoms are difficult for women to comprehend and be understood by others.^7^ Further, they are difficult to manage, negatively affecting the patients’ functioning in their occupational roles.^8^

Another study outlined the fact that work contributes not only to a sense of financial security but also to an enhanced degree of self-identity and better social relationships among cancer survivors.^9^ Work is necessary to provide socioeconomic stability for the aging society. However, previous studies have indicated that cancer survivors have trouble in continuing, or even returning to work following primary treatment, such as chemotherapy and radiation therapy.^9^, ^10^, ^11^, ^12^ Factors impeding cancer survivors’ work retention and their return to work include age, disease stage, treatment, symptoms, and work environment.^11^, ^12^, ^13^ However, little is known about the experiences of and the relationship between symptoms and work-related outcomes among breast cancer survivors (BCS) receiving AET. Therefore, this study aimed to (1) examine the relationship between symptoms and work participation and (2) examine the relationship between symptoms and the overall work impairment (OWI) of BCS receiving AET.

## Methods

### Study design

This study utilized a cross-sectional design. BCS were recruited from a breast surgery outpatient clinic from a university hospital in Tokyo, Japan, between May and December 2019. The research outline was briefly explained to potential participants at their scheduled visit. The researcher was introduced if the patients were interested in the details, and the protocol was further explained to BCS who were interested in participating. After providing informed consent, the participants completed the questionnaires. Participants’ medical information was collected from their medical charts.

The inclusion criteria for this study were as follows: (1) female BCS aged 20–64 years at the time of recruitment; (2) BCS who have been undergoing AET for more than three months and less than five years following surgery, chemotherapy, and radiation therapy as necessary; and (3) BCS with the ability to communicate in Japanese and answer a questionnaire.

Survivors with metastasis or recurrence, those with a history of cognitive/mental disorders, or those who no longer worked because of other health-related problems were excluded from this study.

Sample size calculations were based on the results of previous studies that revealed a prevalence rate of at least one symptom occurring between the working and nonworking groups; the ratio of working and nonworking groups was found to be 6:4, with the significance level of 5% on either side, power established at 80%.

### Measures

#### Work-related outcomes

The participants’ degree of work participation was measured via questions regarding their current employment status. Survivors were questioned on their present employment status (categorized into regular/full-time, contracted/part-time, self-employed, and unemployed groups) and if there was a change since their diagnosis. If changes had occurred, the participant’s employment status during their diagnosis and the reasons behind the changes were recorded. Survivors were classified under the “work participation” category if they were working at the time of the survey or if their employment status had changed between their diagnosis and this survey.

In the employed group, participants’ OWI was assessed using the Japanese version of the Work Productivity and Activity Impairment (WPAI) scale. The WPAI is a six-item questionnaire that assesses respondents’ absenteeism (i.e., the percentage of work time missed due to any health-related problems), presenteeism (i.e., the percentage of impairment while working due to health problems), and the percentage of activity impairment due to health problems.^14^ Participants’ OWI due to health-related outcomes was calculated as absenteeism ​+ ​{(1-absenteeism) ​× ​presenteeism/10}. Higher scores indicated a greater degree of work impairment. The participants were further divided into either high or low OWI groups, based on their score in relation to the median OWI score across this sample group.

In addition, answers regarding work-related factors, including perceived workplace support (e.g., support received from their supervisors or colleagues) and the availability of other tangible support (e.g., whether they received paid time off for any medical appointments or flex time), were obtained using yes or no questions.

#### Symptom burdens

Endocrine therapy-related symptoms were assessed using the Japanese version of the Patient-Reported Outcome Common Terminology Criteria for Adverse Events (PRO-CTCAE). Based on a literature review, the following symptoms were measured: vasomotor symptoms (including hot flashes and increased sweating), joint pain, cognitive problems (including concentration and memory problems), and insomnia. The severity of these symptoms in the previous week was rated on a categorical scale (scale options included “none,” “mild,” “moderate,” “severe,” or “very severe”).^15^^,^^16^

Fatigue was measured using the Functional Assessment of Chronic Illness Therapy-Fatigue (FACIT-F). The FACIT-F is a 13-item questionnaire that assesses respondents’ self-reported fatigue experienced in the previous week.^17^^,^^18^ Each item is rated on a scale from zero (not at all) to four (very much), with the maximum possible score of 52. Lower scores indicate that the respondent experiences severe fatigue. Fatigue was categorized as either “high” or “low” based on an individual’s score (lower or higher, respectively) than the sample’s median score of 44.

Distress was measured using the K6, which is a six-item questionnaire developed to assess nonspecific distress.^19^ Higher scores in this scale indicate severe psychological distress. The optimal cut-off point for this scale in Japan is estimated to be four or five. The Japanese version of the K6 is highly reliable.^20^

All symptom scores were categorized into either a high or low symptom burden based on the moderate or high degree of symptom severity (PRO-CTCAE), the established cut-off points (K6), or the median of this sample (FACIT-F).

#### Survivor characteristics

The demographic information that was collected included participants’ age, marital status, living status, and education. Participants’ medical information was obtained through a review of their medical charts, which included the duration post the initial diagnosis, the type of surgery they underwent, if they received radiotherapy and/or chemotherapy, the type of endocrine therapy they received, and the duration since they began with the endocrine therapy.

### Data analysis

Descriptive statistics were used to characterize each participant and describe the distribution of all the study’s variables. Their sociodemographic, as well as medical characteristics and symptom burdens, were compared between the participant groups (groups of work participation and high or low OWI) using Mann–Whitney U test for all continuous variables. Fisher’s exact test was used for all the categorical variables.

A multiple logistic regression analysis determined the relationship between respondents’ work participation and the number of their symptom burdens. We controlled known factors related to cancer survivors’ work participation, including their age (continuous), duration since their initial diagnosis (continuous), chemotherapy treatment (yes or no), and marital status (either being married or unmarried). A similar analysis was then performed to evaluate the relationship between having a high OWI (vs. having a low OWI) and the number of symptom burdens while controlling for the above-mentioned factors. A two-tailed *P* ​< ​0.05 was regarded as statistically significant.

The data were analyzed using Statistical Package for Social Sciences (SPSS) software, version 26.0.

### Ethical considerations

This study was reviewed and approved by the appropriate Institutional Review Boards. Registration number: UMIN000036742. Written consent of the participants was obtained.

## Results

We approached 144 potential eligible BCS, and four declined to participate. Thus, the number of BCS who participated in total was 140, and all participants completed the questionnaires. The median age of this sample was 51 years (range: 30–64), with the median duration since their initial diagnosis being 26.0 months (range: 5–61). The majority of the participating BCS were married (70.0%) and lived with their family/partner (84.3%). The participants who received chemotherapy were 46 (32.9%), and 80 (57.1%) received radiotherapy. More than half of the participants (*n* ​= ​91, 65.0%) were receiving Tamoxifen.

In this sample, the majority of survivors (*n* ​= ​111, 79.3%) were employed, while 29 (20.7%) were unemployed at the time of the survey, and there were not any significant changes since their initial diagnosis (112 were employed, 28 were unemployed). More than half of the respondents (55.0%) were not regular/full-time workers. In the employed group, the majority (*n* ​= ​96, 86.5%) reported no change in their employment status since the diagnosis, eleven (9.9%) experiencing an upward change in their employment status (i.e., having gone from being unemployed to employed; going from being a contract/part-time worker to regular/full-time), while four (3.6%) experiencing a downward change (i.e., going from being a regular/full-time to being contact/part-time). When compared to the nonworking survivors, the working BCS were younger (with a median age of 50 compared to 52 years, *P* ​= ​0.044), unmarried (35.1% vs. 10.3%, *P* ​= ​0.011), lived alone (19.8% vs. 0%, *P* ​= ​0.008), received chemotherapy (39.6% vs. 6.9%, *P* ​= ​0.001), and had a relatively longer duration pass since their initial diagnosis (a median of 28 vs. 20 months, *P* ​= ​0.035) (Table 1).

**Table 1:** Sample characteristics stratified by employed status and high or low OWI.

Item	Total (*n* ​= ​140)	Not employed (*n* ​= ​29)	Employed (*n* ​= ​111)	*P* value	Employed (*n* ​= ​110)^a^		
					Low OWI (*n* ​= ​53)	High OWI (*n* ​= ​57)	*P* value
Age (years), median (range)	51 (30–64)	52 (37–64)	50 (30–64)	0.044∗	50 (35–61)	50 (30–64)	0.919
Marital status, *n* (%)							
Married	98 (70.0%)	26 (89.7%)	72 (64.9%)	0.011∗	34 (64.2%)	37 (64.9%)	1.000
Single	42 (30.0%)	3 (10.3%)	39 (35.1%)		19 (35.8%)	20 (35.1%)	
Living status, *n* (%)							
Living with family/partner	118 (84.3%)	29 (100.0%)	89 (80.2%)	0.008^∗^	41 (77.4%)	47 (82.5%)	0.634
Living alone	22 (15.7%)	0 (0.0%)	22 (19.8%)		12 (22.6%)	10 (17.5%)	
Education, *n* (%)							
Less than junior college	80 (57.1%)	18 (62.1%)	62 (55.9%)	0.674	31 (58.5%)	30 (52.6%)	0.569
Undergraduate or more	60 (42.9%)	11 (37.9%)	49 (44.1%)		22 (41.5%)	27 (47.4%)	
Chemotherapy, *n* (%), Yes	46 (32.9%)	2 (6.9%)	44 (39.6%)	0.001∗	17 (32.1%)	27 (47.4%)	0.121
Radiotherapy, *n* (%), Yes	80 (57.1%)	20 (69.0%)	60 (54.1%)	0.206	31 (58.5%)	29 (50.9%)	0.449
Endocrine therapy, *n* (%)							
Tamoxifen	91 (65.0%)	16 (55.2%)	75 (67.6%)	0.332	38 (71.7%)	36 (63.2%)	0.619
Aromatase Inhibitors	38 (27.1%)	11 (37.9%)	27 (24.3%)		11 (20.8%)	16 (28.1%)	
＋LHRH	11 (7.9%)	2 (6.9%)	9 (8.1%)		4 (7.5%)	5 (8.8%)	
Time since diagnosis in months, median (range)	26 (5–61)	20 (7–57)	28 (5–61)	0.035∗	30 (7–56)	26 (5–61)	0.654
Time since starting endocrine therapy in months, median (range)	22 (4–55)	18 (6–53)	23 (4–55)	0.091	24 (4–53)	22 (4–55)	0.430
Employment status (*n* ​= ​111), *n* (%)							
Regular/full time	50 (45.0%)				24 (45.3%)	25 (43.9%)	1.000
Contact/part time	61 (55.0%)				29 (54.7%)	32 (56.1%)	
Perceived workplace support, *n* (%)	71 (64.0%)				34 (64.2%)	36 (63.2%)	1.000
Availability of tangible support, *n* (%)	62 (55.9%)				29 (54.7%)	32 (56.1%)	1.000

OWI: Overall Work Impairment; LHRH: Luteinizing hormone-releasing hormone agonist.

∗Statistically significant (*P* ​< ​0.05).

a One employed survivor who was taking sick leave at the time of the survey was excluded.

In the employed group, one working survivor who was on sick leave was excluded from the OWI analysis. The median of the OWI was 10 (range 0–87.6). Table 1 shows the detailed demographic and medical characteristics as stratified by participants’ work participation and high or low OWI. Among the employed survivors, 71 (64.0%) reported a perceived degree of workplace support, with 62 (55.9%) reporting access to a degree of tangible support. There was no significant difference between high or low OWI survivors regarding the median working hours per week (40 vs. 35 ​h, *P* ​= ​0.324) and absenteeism (0 vs. 0, *P* ​= ​0.051), except presenteeism (0 vs. 20, *P* ​≤ ​0.001).

Table 2 displays the symptom burdens as stratified by respondents’ work participation and OWI. The working BCS receiving AET in this study reported having a greater number of symptom burdens than their nonworking counterparts (median number of high symptom burdens was 2.0 vs. 1.0, respectively). In particular, the working BCS reported having a higher symptom burden in terms of hot flashes and problems with their concentration and memory when compared to their nonworking counterparts (Table 2). Across 110 working BCS receiving AET, both the prevalence rates and the median number of high symptom burdens were significantly higher in the high OWI group than within the low one (3.0 vs 0). Moreover, fatigue had the highest prevalence (70.2%) among all the symptoms in the high OWI group.

**Table 2:** Prevalence rates and median number of symptom burdens stratified by employed status and high or low OWI.

Item	Total (*n* ​= ​140)	Not employed (*n* ​= ​29)	Employed (*n* ​= ​111)	*P* value	Employed (*n* ​= ​110)^a^		
					Low OWI (*n* ​= ​53)	High OWI (*n* ​= ​57)	*P* value
Symptom burdens (High symptom severity), *n* (%)							
Hot flashes	42 (30.0%)	4 (13.8%)	38 (34.2%)	0.040∗	12 (22.6%)	26 (45.6%)	0.016∗
Increased sweating	41 (29.3%)	6 (20.7%)	35 (31.5%)	0.360	12 (22.6%)	23 (40.4%)	0.065
Joint pain	31 (22.1%)	6 (20.7%)	25 (22.5%)	1.000	5 (9.4%)	20 (35.1%)	0.001∗
Concentration problems	25 (17.9%)	0 (0.0%)	25 (22.5%)	0.002∗	2 (3.8%)	22 (38.6%)	≤0.001∗
Memory problems	21 (15.0%)	0 (0.0%)	21 (18.9%)	0.007∗	2 (3.8%)	18 (31.6%)	≤0.001∗
Insomnia	32 (22.9%)	7 (24.1%)	25 (22.5%)	0.809	3 (5.7%)	22 (38.6%)	≤0.001∗
Fatigue	62 (44.3%)	13 (44.8%)	49 (44.1%)	1.000	8 (15.1%)	40 (70.2%)	≤0.001∗
Distress	37 (26.4%)	4 (13.8%)	33 (29.7%)	0.100	7 (13.2%)	25 (43.9%)	0.001∗
Number of the high symptom burden, median (range)	1.0 (0–8)	1.0 (0–6)	2.0 (0–8)	0.044∗	0 (0–6)	3.0 (0–8)	≤0.001∗

OWI: Overall Work Impairment.

^∗^Statistically significant (*P* ​< ​0.05).

a One employed survivor who was taking sick leave at the time of the survey was excluded.

The logistic regression analyses on work participation revealed a positive association between attaining chemotherapy (odds ratio (OR) ​= ​6.02, 95% CI, 1.27–28.60, *P* ​= ​0.024) and being unmarried (OR ​= ​4.07, 95% CI, 1.09–15.18, *P* ​= ​0.036). Symptom burdens were not significantly associated with respondents’ employment status (Table 3). The logistic regression analyses on participants’ OWI revealed that there is a statistically significant association of high OWI with the number of high symptom burdens (OR ​= ​2.14, 95% CI, 1.58–2.89, *P* ​≤ ​0.001) and the duration since their diagnosis (OR ​= ​0.95, 95% CI, 0.91–0.99, *P* ​= ​0.019) (Table 4).

**Table 3:** Logistic regression of work participation (*n* ​= ​140).

Item	Odds Ratio	95% CI		*P* value
Age	0.935	0.863	1.012	0.097
Time since diagnosis	1.017	0.982	1.053	0.344
Chemotherapy	6.022	1.268	28.603	0.024∗
Not married	4.073	1.093	15.184	0.036∗
The number of high symptom burden	1.230	0.958	1.578	0.104

∗Statistically significant (*P* ​< ​0.05).

CI: Confidence interval.

**Table 4:** Logistic regression of overall work impairment (*n* ​= ​110).

Item	Odds Ratio	95% CI		*P* value
Age	1.017	0.939	1.100	0.686
Time since diagnosis	0.952	0.914	0.992	0.019∗
Chemotherapy	2.436	0.847	7.012	0.099
Not married	1.162	0.430	3.137	0.768
The number of high symptom burden	2.137	1.579	2.892	≤0.001∗

∗Statistically significant (*P* ​< ​0.05).

CI: Confidence interval.

## Discussion

### Relationship between work participation and symptom burdens

In this study, 79% of BCS were employed, with almost no change in their employment status since their diagnosis. It was also identified that working BCS receiving AET experienced greater hot flashes and cognitive problems than their nonworking counterparts. Furthermore, greater than 44% of working BCS reported high severity of fatigue, although this did not seem affected by their working status. This demonstrates that BCS receiving AET continued to work despite experiencing these symptoms.

In previous studies, symptoms such as fatigue and cognitive problems were found to be significantly associated with nonemployment,^11^, ^12^, ^13^^,^^21^ which was inconsistent with our findings. Having a degree of financial need is a probable reason the participants continue to work despite experiencing these symptom burdens. It plays a significant role in the association between BCS work participation and their single status. A systematic review of BCS’ returning to work concluded that being younger, single, divorced, or widowed facilitated their return to work due to a greater degree of financial insecurity.^12^ A previous study also identified that chemotherapy results in a greater financial burden on cancer survivors.^22^ Our findings reveal a higher proportion of working BCS in this study. They are relatively young, single, and have a history of chemotherapy, which are all suitably associated with a higher degree of financial need.

Workplace support is another probable reason for BCS continuing to work despite facing these symptoms. In this study, more than 60% of the participating BCS felt they had received support from their workplaces, and greater than half of them utilized tangible support such as sick leave and flex time. It has been emphasized that a supportive work environment and workplace accommodations are necessary to promote BCS’ employment continuation.^9^, ^10^, ^11^, ^12^

These results suggest that continuous support for working BCS not only from healthcare professionals but also from employers may be necessary throughout the long-term AET period to sustain their work life.

### Relationship between overall work impairment and symptom burdens in working Breast Cancer Survivors

OWI was calculated in this study based on participants’ absenteeism and presenteeism, with most of these variables among participants being dominated by presenteeism (measured as perceived decreased productivity). Almost all symptoms were associated with high OWI, with the results revealing the manner in which symptoms adversely affect BCS’ perceived productivity. Previous studies have also reported that hot flashes, fatigue, cognitive impairments, and distress are all symptoms that interfere with BCS’ productivity, which is consistent with our findings.^10^^,^^23^, ^24^, ^25^ In addition, these symptoms reduce survivors’ satisfaction and their capacity to function within their work.^26^ This adversely affects their work life and worsens their work-related stress, and is associated with changes in identity and role function, diminished self-confidence, a sense of social isolation.^27^ Observing the sense of decreased productivity among survivors reflects a sense of dissatisfaction and a loss of self-confidence. This is due to the inability to perform with the same intensity as before the cancer diagnosis.

In addition, the logistic regression analyses on participants’ OWI revealed that participants who reported an increased number of symptom burdens had twice the odds of reporting high OWI as their counterparts. This suggests that having multiple symptoms increases their work-related difficulties. Previous studies on symptom clusters found that survivors who experienced multiple co-occurring symptoms tended to not work compared to others who did not have multiple symptoms^28^, the former reporting an even lower QOL.^29^ Survivors who experience multiple symptoms require more support due to their high-risk conditions that result in a suboptimal quality of work life.

Notably, there was no significant association between OWI and the history of chemotherapy of BCS receiving AET in this study. When compared to chemotherapy, endocrine therapy is often recognized as an increasingly tolerable treatment. However, for some BCS receiving AET, experiencing multiple co-occurring symptoms may affect their overall long-term QOL, especially in terms of their work life. A qualitative study of BCS receiving AET revealed that they were confused by the wide range of unexpected endocrine therapy-related symptoms and their impact, unclear on the underlying reasons of these symptoms, as well as experiencing difficulties that others cannot comprehend.^7^ Previous studies on the return-to-work process of cancer survivors suggest that excessive expectations of their supervisors and colleagues, as well as excessive protective reactions, resulted in a significant burden.^10^^,^^26^^,^^30^ The perceived decrease in productivity reflects the present situation of survivors, who feel the impact of these unexpected symptoms, as they attempt a return to their daily lives and struggle with being not understood by others.

### Limitations

This study has a few limitations. First, because it uses a cross-sectional design, we were unable to explain the causal relationship between symptoms and endocrine therapy or between these symptoms and work participation or OWI. Further, it is unclear if the endocrine therapy is solely responsible for these symptoms. Hot flashes may also manifest due to premature menopause caused by chemotherapy. Cognitive problems can also be indistinguishable; unclear whether caused due to endocrine therapy or a “chemobrain.” Additionally, the sample was heterogeneous. Although only BCS receiving AET were recruited, time intervals since the diagnosis and the history of the adjuvant treatment were diverse. However, our findings remain clinically significant as they outline BCS’ symptomatic experiences and their impact on work during AET.

Finally, the survey was conducted at one urban hospital in Japan; therefore, generalization of these results is difficult. In addition, because the survey was conducted on BCS within three months to five years after the initiation of AET, survivors who discontinued endocrine therapy were not included.

### Implications for practice and research

Healthcare professionals need to inform both survivors receiving the AET and their employers of the multiple long-term symptoms and their impact on the BCS’ work productivity. While assessing them continuously and regularly, they should provide symptom management support throughout the period of the AET. In addition, survivors who experience moderate or severe symptoms face a decrease in their work productivity. Thus, workplace and tangible support are needed according to their individual backgrounds and needs. Further research is needed to evaluate effective symptom management and workplace support practices, as well as to develop effective care programs that enhance the quality of the work life of BCS receiving AET.

## Conclusions

BCS receiving AET were found to continue or return to work while experiencing multiple symptom burdens. However, survivors experiencing these long-term symptoms were negatively affected in their work life. Greater support from healthcare professionals and employers is needed to improve the quality of work life in BCS receiving AET.

## Funding

This study was funded by 10.13039/501100001697Keio University Doctoral Student Grant-in-Aid Program. Partial financial support was received from Kitasato University School of Nursing, Japan.

## Declaration of competing interest

None declared.
